# Transverse Sinus Hypoplasia as a Differential Diagnosis for Cerebral Vein Thrombosis: A Case Report

**DOI:** 10.1002/ccr3.71199

**Published:** 2025-10-07

**Authors:** Hamidreza Ashayeri, Fateme Javanali Azar, Hadi Vahedi, Ariodad Ranji‐Amjad, Ali Jafarizadeh

**Affiliations:** ^1^ Student Research Committee Tabriz University of Medical Sciences Tabriz Iran; ^2^ Liver and Gastrointestinal Diseases Research Center Tabriz University of Medical Sciences Tabriz Iran; ^3^ Nikookari Eye Center Tabriz University of Medical Sciences Tabriz Iran

**Keywords:** cerebral vein thrombosis, deep vein thrombosis, headache, magnetic resonance venography, transverse sinus hypoplasia

## Abstract

We present a 43‐year‐old female who had a history of deep vein thrombosis (DVT) and pulmonary emboli and was diagnosed with DVT. The patient reported a headache and underwent an emergency CT scan, which revealed no intracranial hemorrhage (ICH). To evaluate the possibility of CVT, the patient underwent magnetic resonance venography (MRV), which showed a defect in the right transverse sinus. To differentiate between CVT and sinus hypoplasia, MRV with gadolinium contrast was done, which revealed a right hypoplastic transverse sinus. This educational case highlights the importance of detecting hypoplastic transverse sinus from CVT, especially in patients with a possible hypercoagulative state. This differentiation will save patients from unnecessary treatment.

**Summary**

- Headaches in a patient with a hypercoagulative state and receiving anticoagulant treatment can be due to the ICH or CVT.
- Transverse sinus hypoplasia can mimic CVT in MRV, and differentiating them can reduce the need for unnecessary treatment.
- The hypoplasia of the right transverse sinus is rare compared to the left side, which can mislead clinicians.
- Sigmoid notch asymmetry, jugular foramen size difference in CT are some useful signs in predicting the presence of hypoplastic central sinus.
- Blooming sign and brush sign on SWI sequence are accurate ways to identify CVT and cortical vein thrombosis in MRI.

## 1 Introduction

Headaches are among the most common neurological complaints. There are a variety of conditions that can present with headaches. Some of these conditions, such as migraines, are benign. However, some of them, such as subarachnoid hemorrhage or cerebral venous thrombosis (CVT), can be fatal. CVT is a condition in which a clot obscures the normal blood flow in the cerebral sinus and veins. CVT can cause headaches, seizures, and a variety of focal neurologic deficits [1]. The annual incidence of CVT is reported to be 13.9 to 20.2 per 1 million population, and women in the 18–44 age range are the most at‐risk population group [2]. However, cerebral sinus hypoplasia is a condition that can mimic CVT in imaging. Here, we present a female case with DVT who developed a headache during the hospital stay. At first, imaging suggested CVT, but the additional imaging revealed right transverse sinus hypoplasia.

## 2 Case History and Examination

A 43‐year‐old female patient with a history of DVT and pulmonary embolism (PE) presented to the emergency department with a complaint of unilateral leg swelling. The previous DVT + PE happened 19 months before the current presentation. The patient's previous DVT + PE occurred after 10 days of using oral contraceptive pills (OCP), and the patient has been under anticoagulant therapy (apixaban) since then and hasn't used OCP anymore. The patient reports that recently, she has been using apixaban irregularly.

Due to the lower limb circumference difference between the left and right legs (left leg: 37 cm, right leg: 33 cm), the patient underwent a limb sonography and was hospitalized with the diagnosis of DVT. The patient didn't have any complications of dyspnea or chest pain. The patient's heart rate was 80, with a blood pressure of 120/80 and oxygen saturation of 94%. Unfractionated heparin (UFH) was started for the patient to treat DVT. Due to the recurrence of DVT, an investigation of the cause of the hypercoagulative state was performed. The protein C, S, and factor V Leiden were reported to be 133, 67, and 2.74 (reference range: 70–140, 60–135.8, 2.18–3.38). IgG and IgM‐β2 glycoprotein Ab were also in the normal range (IgG was 3.32, and IgM was 0.6 with a reference range of < 5 U/mL).

On the second day, the patient reported a headache in the frontal region, which worsened when she coughed. She described her pain to be 6 on a scale of 1 to 10 and felt nausea but had no vomiting. The patient was alert and oriented. She didn't have any history of seizures, ophthalmoplegia, paresthesia, decreased vision, or focal neurologic deficit in her neurologic examination. In the ophthalmoscopy examination, no papilledema was seen. The headaches were unresponsive to therapy with acetaminophen. Despite adequate dosing, the UFH was changed to enoxaparin on the third day since the activated partial thromboplastin time (aPTT) didn't reach the desired therapeutic range.

## 3 Methods

### 3.1 Investigations

**FIGURE 1:**
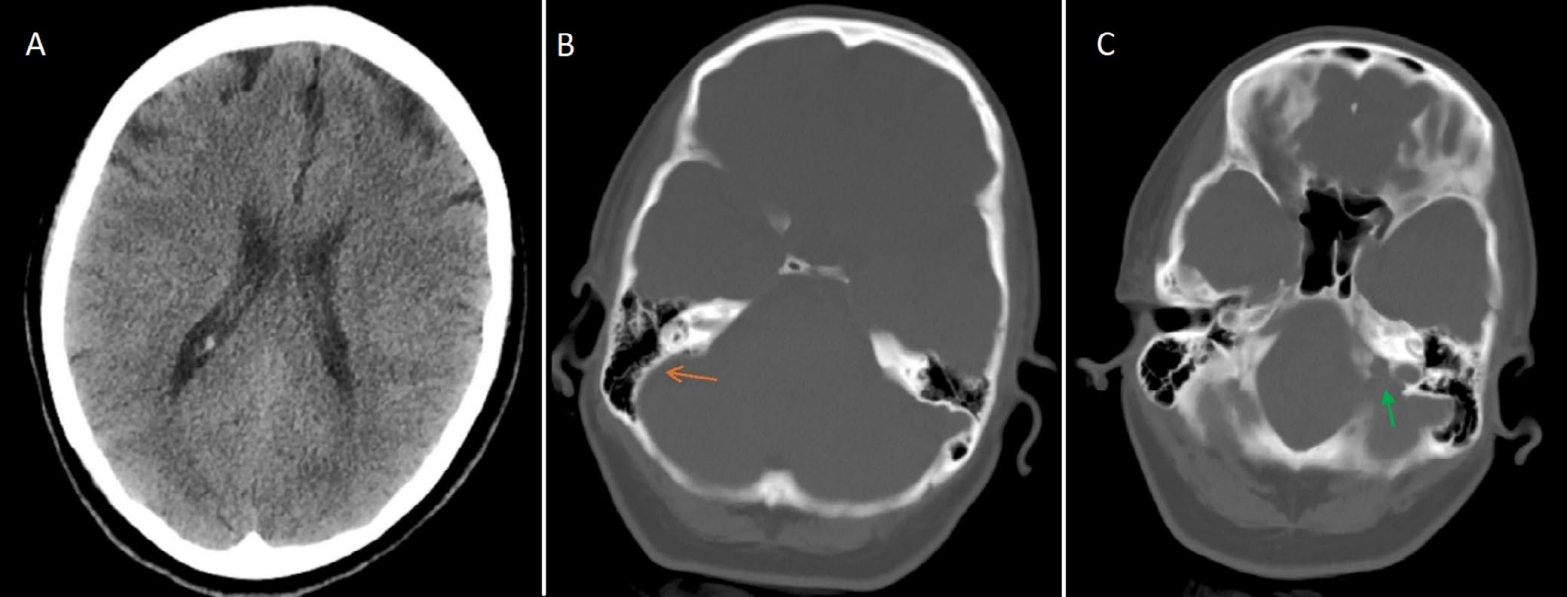
Brain computed tomography (CT). (A) soft tissue window, (B) and (C) bone window. No signs of intracranial hemorrhage or ventriculomegaly. The orange flash in the right image shows a lack of a sigmoid notch on the right side. The green arrow indicates the left jugular canal, which is absent on the right side.

Due to concerns of intracranial hemorrhage (ICH), the patient underwent an emergency computed tomography (CT). There were no signs of intracerebral hemorrhage or ventriculomegaly on the CT. Figure 1 and Video S1 represent the brain CT of the patient. The CT showed asymmetry of the sigmoid notch and jugular foramen on the right side, indicating the possibility of sinus hypoplasia.

**FIGURE 2:**
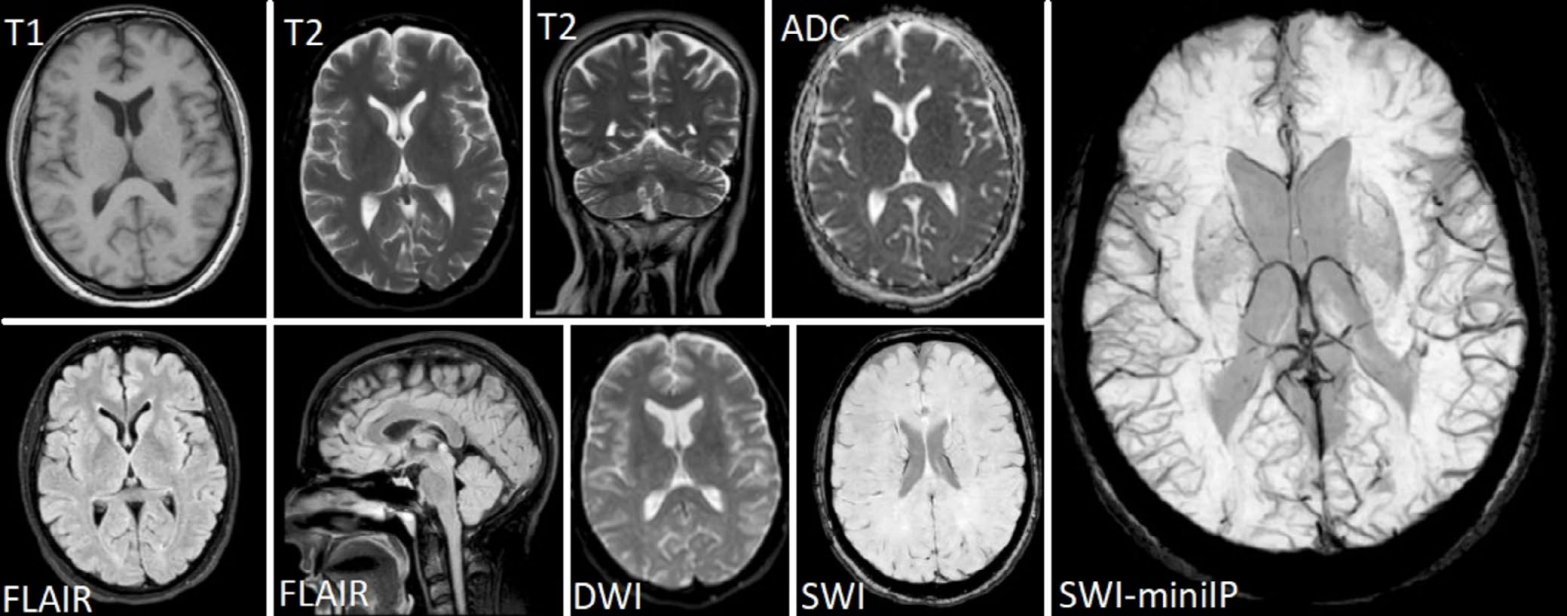
Brain magnetic resonance of the patient. The MRI of patients showed no signs of infarction, chronic hemorrhage, or ventriculomegaly. The SWI‐miniIP sequence shows no blooming artifact. ADC, apparent diffusion coefficient, DWI, diffusion‐weighted imaging, FLAIR, fluid‐attenuated inversion recovery, SWI, susceptibility‐weighted imaging.

Due to concerns about CVT, the patient underwent brain magnetic resonance imaging (MRI) and magnetic resonance venography (MRV). No ischemic or hemorrhagic lesion on MRI or blooming artifact or brush sign on SWI‐miniIP was seen. However, MRV revealed filling defects in the right transverse and sigmoid sinuses. Figure 2 presents the patient's MRI.

### 3.2 Differential Diagnosis

The intensity of the headache also decreased significantly. Due to the incongruity between the imaging and physical examination, the possibility of right transverse sinus hypoplasia was considered. To differentiate between CVT and sinus hypoplasia, patients underwent MRV with gadolinium contrast.

### 3.3 Outcome

**FIGURE 3:**
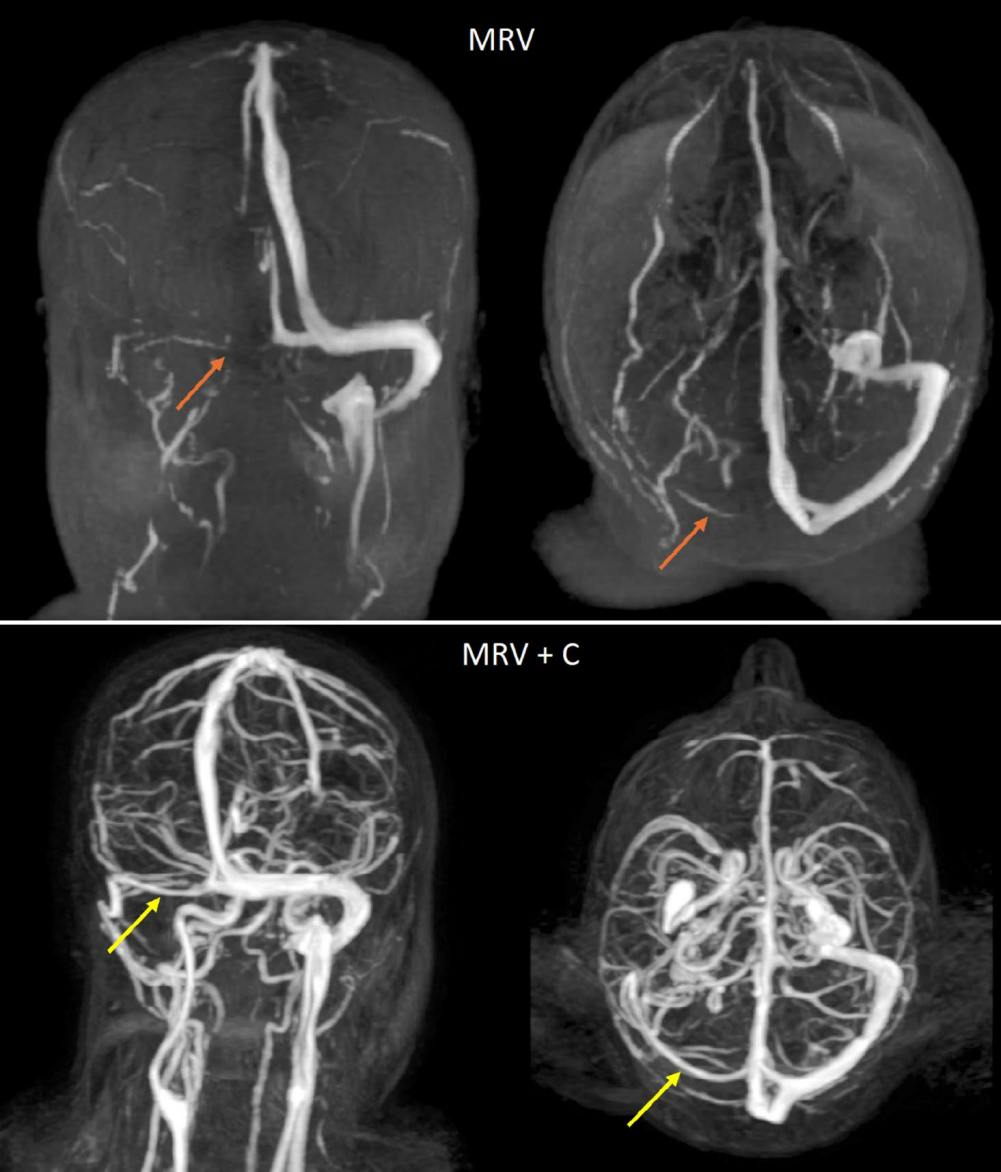
The top row shows the patients' magnetic resonance venography (MRV). There is a defect in the right transverse sinus. The orange arrow points to the possible position of the right transverse sinus. The row below shows the MRV with gadolinium contrast of the patient. The yellow arrow points to the hypoplastic right transverse sinus.

The imaging result revealed the right transverse sinus hypoplasia. On the fourth day, the left leg circumference was 34 cm, and the edema had resolved. After the headache resolved, the patient was discharged on day seven, with the recommendation to use anticoagulant therapy regularly. Figure 3 and Video S2 show the patient's MRV and MRV with gadolinium contrast.

## 4 Discussion

Hypoplasia of the transverse sinus is defined as the diameter of the transverse sinus being less than 50% of the superior sagittal sinus [3]. The transverse sinus hypoplasia is a common anatomical variation seen in 20%–39% of the population [4]. This common variation can mimic the appearance of CVT. CVT most commonly occurs in the sagittal and transverse sinuses, and in 18%–50% of cases, multiple sinuses are involved [5]. Hypoplasia and aplasia of the left transverse sinus are more common than the right transverse sinus [6]. Higher rates of headaches and migraine were reported in patients with transverse sinus hypoplasia due to the possibility of impaired venous return [7, 8]. In a rare case report by Parsian et al. [9], bilateral transverse sinus hypoplasia was associated with a headache in a pregnant patient. These imply that this situation should not be underscored in importance, and this should be considered to avoid unnecessary treatments. Sigmoid notch asymmetry on CT can indicate the presence of hypoplastic transverse sinus with a sensitivity/specificity of 91%/86% [10]. The brush sign in the SWI sequence is reported to have a sensitivity/specificity of 93%/100% in cortical vein thrombosis detection [11].

In our case, the patient had a headache and received anticoagulant therapy. So, the first suspected cause was hemorrhage, which a normal CT rules out. Due to the possibility of a hypercoagulative state, the second suspicion was CVT. After ruling out the possibility of CVT, the diagnosis of right transverse sinus hypoplasia was made. Despite the rarity of right sinus hypoplasia, it should always be considered after ruling out emergency and life‐threatening causes such as CVT.

## Author Contributions

**Hamidreza Ashayeri:** conceptualization, investigation, visualization, writing – original draft, writing – review and editing. **Fateme Javanali Azar:** investigation, writing – original draft, writing – review and editing. **Hadi Vahedi:** writing – original draft, writing – review and editing. **Ariodad Ranji‐Amjad:** writing – original draft, writing – review and editing. **Ali Jafarizadeh:** conceptualization, project administration, visualization, writing – original draft, writing – review and editing.

## Ethics Statement

This study was conducted in accordance with the principles outlined in the Declaration of Helsinki.

## Consent

Written informed consent for the publication of any accompanying images was obtained from the patient.

## Conflicts of Interest

The authors declare no conflicts of interest.

## Supporting information


**Video S1:** Brain CT of the patient, indicating the absence of the right sigmoid notch.

**Video S2:** Brian's magnetic resonance venography (MRV) of the patient, showing a defect in the right transverse sinus, and brian MRV of the patient with gadolinium contrast, confirming the diagnosis of transverse sinus hypoplasia.

## Data Availability

The datasets supporting the conclusions of this article are included within the article and its additional files.
